# Variability of Rheotaxis Behaviors in Larval Bullfrogs Highlights Species Diversity in Lateral Line Function

**DOI:** 10.1371/journal.pone.0166989

**Published:** 2016-11-21

**Authors:** Erika E. A. Brown, Andrea Megela Simmons

**Affiliations:** Department of Cognitive, Linguistic & Psychological Sciences, Brown University, Providence, Rhode Island, 02912, United States of America; Virginia Commonwealth University, UNITED STATES

## Abstract

The morphology and distribution of lateral line neuromasts vary between ecomorphological types of anuran tadpoles, but little is known about how this structural variability contributes to differences in lateral-line mediated behaviors. Previous research identified distinct differences in one such behavior, positive rheotaxis towards the source of a flow, in two tadpole species, the African clawed frog (*Xenopus laevis*; type 1) and the American bullfrog (*Rana catesbeiana*; type 4). Because these two species had been tested under different flow conditions, we re-evaluated these findings by quantifying flow-sensing behaviors of bullfrog tadpoles in the same flow field in which *X*. *laevis* tadpoles had been tested previously. Early larval bullfrog tadpoles were exposed to flow in the dark, in the presence of a discrete light cue, and after treatment with the ototoxin gentamicin. In response to flow, tadpoles moved downstream, closer to a side wall, and higher in the water column, but they did not station-hold. Tadpoles exhibited positive rheotaxis, but with long latencies, low to moderate accuracy, and considerable individual variability. This is in contrast to the robust, stereotyped station-holding and accurate rheotaxis of *X*. *laevis* tadpoles. The presence of a discrete visual cue and gentamicin treatment altered spatial positioning and disrupted rheotaxis in both tadpole species. Species differences in lateral-line mediated behaviors may reflect differences in neuromast number and distribution, life history, or perceptual salience of other environmental cues.

## Introduction

In their natural environments, aquatic anamniotes are exposed to current flow, whether from discrete, localized disturbances (such as in still waters) or as a change from a moving background (such as in flowing waters). Changes in ambient current can signal the presence of predators, prey, or physical obstacles, and detection of these changes can be critical for orientation, navigation, feeding, and defensive behaviors. The mechanosensory lateral line system is specialized to detect both steady and pulsatile water flows. Its structure and functioning have been examined extensively in fishes active at different water depths and in a variety of habitats [1,2].

Aquatic anuran amphibians also possess a lateral line, but, compared to the wealth of information available on this sensory system in fishes, considerably less is known about its operation. Unlike in fishes, in anurans the lateral line sensors, neuromasts, are not easily divided into superficial and canal types [3]. Neuromasts vary considerably in their number, size, and organization between tadpoles classified into four different ecomorphological types [4,5]. How ecological and morphological differences translate into behavioral differences in lateral line function has not been fully explored. Quantitative analyses of flow sensing behaviors in anurans have been limited to two species, the African clawed frog, *Xenopus laevis* (type 1), during larval and adult stages [6–13] and the bullfrog, *Rana* (formerly *Lithobates*) *catesbeiana* (type 4), during larval stages [14]. Tadpoles of these two species differ in their propensity to exhibit rheotaxis, a standard metric of lateral line function which in fishes is mediated by superficial neuromasts [1,15]. *X*. *laevis* tadpoles show robust positive rheotaxis (orientation towards the direction of the flow source) [12,13] while bullfrog tadpoles of comparable developmental stages are more randomly and more variably oriented [14]. These differences in behavior may arise from several variables related to ecomorphological type, including neuromast organization, ecologies, foraging and predator avoidance behaviors, and life history.

It is also possible that the absence of positive rheotaxis by bullfrog tadpoles [14] reflects differences in experimental conditions, such as the characteristics of the particular flow field used in that experiment, rather than species differences related to ecomorphological type. In this experiment, we test this hypothesis by examining flow sensing behaviors of bullfrog tadpoles in the same flow field as used previously with *X*. *laevis* [13]. We also examine the impact on rheotaxis of two additional experimental manipulations—the presence of visual cues and treatment with gentamicin, an ototoxin that can damage neuromasts [1]. Discrete visual cues disrupt rheotaxis in larval *X*. *laevis* [13]; the influence of vision on rheotaxis in fishes varies with species, but in some testing conditions can guide, rather than disrupt, orientation behaviors [1, 16–19]. Gentamicin administration disrupts rheotaxis in larval *X*. *laevis* [13] as well as in fishes [20,21]. Combining these manipulations allows us to assess the salience of visual cues on rheotaxis both when the lateral line is functioning and when it has been damaged. We hypothesized that bullfrog tadpoles would not show the robust, accurate rheotaxis exhibited by *X*. *laevis* tadpoles, even when tested under the same flow conditions, and that their behaviors would be disrupted by visual cues and by gentamicin treatment.

## Materials and Methods

### Ethics statement

Experimental procedures were approved by the Brown University Institutional Animal Care and Use Committee and are consistent with guidelines outlined in the Guide for the Care and Use of Laboratory Animals (eighth edition, National Research Council of the National Academies of Sciences USA).

### Animals

Bullfrog tadpoles were obtained from commercial suppliers (Dozier Lester, Duson, LA; Arizona Aquatic Gardens, Oro Valley, AZ). Tadpoles were housed in groups in polycarbonate aquaria filled to a depth of 20cm with treated housing water (reverse osmosis water with added salts to maintain pH around 7.5). About one-third of the aquaria water was replaced every other day, and the tanks were kept clear of detritus from elimination products or uneaten food. Tadpoles were fed every other day with a mixture of unsalted cooked spinach and a paste made of trout pellets. The colony room was maintained at a room temperature of 25–28°C on a 12/12 light/dark cycle. After behavioral testing, animals were moved to new housing aquaria for use in other experiments or were euthanized. Tadpoles were staged according to the Gosner [22] staging criteria, which are based on external morphological criteria. Testing was limited to tadpoles between Gosner stages 25–30, classified as early larval tadpoles [23]. Tadpoles in these stages are active, free swimmers, with short globose bodies and long muscular tails (length from snout to tip of tail varying between 48–75 mm), developing hindlimb buds (without foot paddles or toes), and no forelimbs. Lateral line neuromasts and inner ear hair cells are present, but neither middle ear transduction pathways nor an external tympanum have developed [24]. Older tadpoles were not tested because the lateral line begins to degenerate prior to the onset of metamorphic climax.

### Flow tank and flow field

Bullfrog tadpoles were tested in a custom-built recirculating flow tank (based on the design of [25]), identical to that used to test *X*. *laevis* [13]. Tanks of similar design have been used to study rheotaxis in fishes [18, 26, 27]. Dimensions of the tank were 105 cm x 15 cm x 20 cm, with a working area of 68 cm (X dimension, streamwise), 15 cm (Y dimension, crosswise), and 15 cm (Z dimension, depth). The tank was supported on an aluminum stand covered in neoprene cushioning material and supported by a granite table. A motorized impeller with adjustable speed control (Type 245A; Bodine Electric Co., Northfield, IL, USA) mounted on a separate stand near the downstream end of the tank was used to produce and control the water flow. The impeller pumped water through a polyvinylchloride tube (10 cm diameter) located below and extending the full length of the tank to the upstream end, where the water then flowed through a funnel (10 cm diameter) located at the crosswise midpoint, through two adjacent collimators (each 2.5 cm long with 0.5 cm diameter tubes) covered with fine mesh and then into the working area of the tank. The collimators reduced turbulence and produced a more uniform flow. Water flowed through the working area of the tank to the downstream end, through another fine mesh screen to prevent tadpoles from being sucked out of the tank, and then back into the polyvinylchloride tube for recirculation. The tank, stand, and motor were fully enclosed by opaque vinyl to eliminate any stray visual cues. The computer controlling the experiment was located outside of the vinyl-enclosed tank area, with the monitor pointed directly opposite the tank so as not to provide any illumination of the tank itself.

Water flow speeds were set by recording the speed of movements of methylene blue dye throughout the tank produced by the motor at various settings on the motor control dial. Droplets of dye were introduced into the upstream end of the tank at several different water depths, and their movements were videotaped using a high speed infrared-sensitive digital camera (Casio EX-FH100, Tokyo, Japan; 120 frames/s). The average rate of dye movement at a given setting on the motor control dial was calculated (from a mean of three measurements) and used as an estimate of flow speed. By this method, five different flow speeds (2, 4, 6, 8, and 10 cm/s) were identified and marked on the motor control knob; these included the three flow speeds of 6, 8 and 10 cm/s used previously [14]. Pilot experiments indicated that flow speeds of 1 cm/s are near the tadpoles’ threshold for detection while flow speeds of 12 cm/s and faster pushed the tadpoles downstream against the back wall of the tank and often caused injury. The flow field at each flow speed was visualized using digital particle image velocimetry (DPIV), as previously described [13, 28]. In this method, movements of silver-coated glass spheres (mean diameter 12μm, density 1.3 g cm^–3^; conduct-o-fil, Potters Industries, Valley Forge, PA) suspended in the flow field were visualized using a pair of 80mW line lasers (series LBS, Laserglow, Toronto, CA) mounted collinearly above the tank so that they illuminated a single plane. These lasers were moved in 1 cm steps along the plane parallel with the X axis and down the Y axis until the entire tank was sampled (32 total sampling points). Flow fields at each speed are based on analysis of 150 frames of video recording. The flow was measured in sequential cross-sections throughout the tank using custom-written MATLAB (R2010a; Math Works, Natick, MA) code based on the openPIV software package [29], and data were interpolated to produce evenly-spaced measurements across the sampling points. Three-dimensional (3-D) data were obtained by combining velocimetry measurements from the stack of videos. The flow field is relatively laminar in the X dimension throughout most of the Y and Z dimensions, but with boundary layer effects near the edges (at Y = 1, Y = 15, and Z = 1 cm). It is identical to the flow field used to test rheotaxis in *X*. *laevis* [13] (experiments with the two species were conducted concurrently), but it is more spatially homogeneous than that illustrated in [14]. These differences in the flow field resulted from closer spacing of the upstream collimators and the use of a different impeller motor.

Tadpoles’ movements in the tank were recorded using four synchronized, infrared-sensitive digital cameras (BW DSP CCD; Supercircuits, Austin, TX). Two cameras were suspended on a beam located 42 cm above the tank (one at the upstream and one at the downstream end), and two were fixed to an aluminum frame placed 112 cm away from and parallel with the midline of the tank. The four cameras were calibrated for 3D reconstruction by digitizing the positions of 24 calibration points for a direct linear transform (DLT) algorithm [30]. DLT was implemented using a custom-designed MATLAB routine that included code from the DLTdv3 toolkit [31].

### Experimental design and procedure

#### Experiment 1

Experiment 1 was conducted in the dark, with the only light source from infrared LEDs mounted above the tank. Tadpoles (N = 267) were divided into two groups, untreated control animals (N = 158) and gentamicin-treated animals (N = 109). Prior to testing, an individual tadpole was removed from its home aquarium, placed into a new, smaller aquarium, and immersed for 24 hours, either in housing water or in a solution of 0.02% gentamicin sulfate (500 μM; G1914, Sigma, St. Louis, MO). The gentamicin dosage is the same as used in *X*. *laevis* tadpoles [13] and in larval zebrafish (*Danio rerio*; [21]). The treatment itself had no visible, deleterious effects on normal swimming behaviors in the aquarium; no tadpoles died during the immersion period or during testing.

At the end of the 24 hour immersion period, each tadpole was placed for 1 hour in housing water, and was then introduced into the testing tank. An individual tadpole was tested only once. Testing was conducted at the same time each day to minimize circadian effects. Using a small water-filled beaker, a tadpole was released into the center (X = 34 cm in a 68 cm working area) of the tank at the water surface. At the time of release, there was no water flow through the tank. The animal’s movements were recorded for 300 seconds in this No Flow (NF) period. At the end of this 300 second period, the impeller motor was turned on, with the adjustable dial set to one of the five pre-determined speeds. During this With Flow (WF) period, the tadpole’s movements were recorded for another 300 seconds, for a total trial length of 600 seconds. The different flow speeds were tested in the order of 8, 10, 4, 6, and 2 cm/s, with individual tadpoles being randomly assigned to untreated or treated groups within this order.

At the end of testing, the animals were either moved into new housing aquaria for use in other experiments or were processed for visualization of neuromasts using the fluorescent vital dye DASPEI (0.01% solution, 2-(4-(dimethylamino)styryl-)–N-Ethylpyridinium Iodide; Molecular Probes, Life Technologies, Grand Island, NY). DASPEI is taken up by mitochondria in active hair cells [20, 21]. Tadpoles were immersed in the DASPEI solution for 10 minutes in the dark, immediately after behavioral testing. They were then anesthetized in 0.6% buffered tricaine methanesulfonate (MS-222, pH 7.0; Sigma) for 1 minute and placed on a glass microscope slide. Stained neuromasts (dorsal on head, trunk and tail; ventral on head and trunk) were visualized using an Olympus BX-60 fluorescent microscope and DP72 digital camera (Olympus, Melville, NY). Bullfrog tadpole skin is highly pigmented, with numerous chromatophores and iridophores which often also fluoresced. Neuromasts were distinguished from these pigmented cells by their shape and stronger fluorescence. After photographing and counting all stained neuromasts, tadpoles were euthanized by immersion in 0.6% MS-222 for 20 minutes.

#### Experiment 2

Testing proceeded as in Experiment 1, with the addition of discrete visual cues. A white LED flashlight (spectral peaks at 460 nm and 580 nm) was mounted above the tank at a height of Z = 16 cm, either upstream at the source of the flow (Light Upstream; X = 1 cm) or downstream at the location of the impeller (Light Downstream; X = 68 cm). In both locations, the light illuminated a region of about 12cm in the X dimension. Tadpoles (N = 52; untreated, N = 19; treated, N = 33) were individually placed into the tank with the light already illuminated, and the light remained illuminated during both NF and WF periods. All animals were tested at a flow speed of 2 cm/s. Each animal was tested once, at only one light location. Within a given testing day, the light was presented in both locations, counterbalanced across animals.

### Data analysis

Two aspects of tadpoles’ movements are of interest, 3-D spatial position in the tank in XYZ coordinates, and orientation towards the source of the flow. These two aspects need not be correlated; for example, a tadpole could conceivably exhibit rheotaxis at any spatial position in the tank, or only when within a certain area. One effect of current flow might be to passively displace animals downstream; alternatively, animals may actively swim upstream or downstream in the presence of flow, or they may station hold. All of these behaviors might be affected by the integrity of the lateral line system. Both spatial positioning and rheotaxis have been quantified in studies of lateral line function in *X*. *laevis* tadpoles [13] and in several species of fishes [18,27].

To quantify tadpoles’ movements, the outputs of the four digital cameras were multiplexed and sent to a Dell computer. Videos were digitized with Adobe Premiere Pro CS4 (Adobe, San Jose, CA) and stored as.mp4 files for off-line analysis. Using a custom-written MATLAB interface, we conducted computer-assisted manual analysis of the video files. Video frames from all four cameras were generated (one frame every 30 seconds over the entire 600 second trial length) and displayed in quadrants on the computer screen. Through manual user inputs of the location of the tadpole’s head and tail within each frame, the program used DLT to calculate both the tadpole’s spatial position in XYZ coordinates and its orientation heading (the position of the head in degrees relative to the source of the flow) in each 30 second segment. These measurements were digitally stored as Microsoft Excel files. Each video was analyzed separately by two observers, and results were compared for reliability. Any video in which measurements differed by more than 10% was reanalyzed. If reliability of >90% could not be achieved (due to missing frames or experimenter error), then that video was removed from the database.

To analyze spatial position, for each tadpole the means of the 10 position measurements in the NF period (one measurement every 30 seconds for 300 seconds) and the means of the 10 measurements in the WF period (one measurement every 30 seconds for 300 seconds) were calculated separately for X, Y, and Z coordinates. These data were used as input for GLM multivariate repeated measures analysis of variance (ANOVA; v. 22, IBM, Armonk, NY), with treatment (2 levels, untreated and treated) and flow speed (5 levels) as between-subjects variables and flow (2 levels, NF and WF) as the repeated measure. XYZ coordinates are the multivariate dependent measures. The Tukey HSD test was used for paired comparisons. Significance levels were set at P<0.05.

For analysis of rheotaxis, the orientation of each tadpole’s head in circular coordinates with respect to the flow source was calculated using a custom script based on the circular statistics toolbox in MATLAB. The mean circular orientation for each tadpole was calculated over the NF period (circular mean of 10 measurements) and over the WF period (circular mean of 10 measurements), so that each data point is the mean of the mean bearing. The statistical significance of the mean orientation vectors in NF and WF over all tadpoles was analyzed using the modified Rayleigh statistic, u [32], to test the hypothesis that tadpoles were oriented upstream towards the source of the flow (positive rheotaxis, orientation towards 0^0^). Consistent with the criterion used in our previous experiments [13, 14], significance levels of P<0.001 were used to reject the null hypothesis of random orientation. Mean vector strength (the length of the orientation vector, varying between 0 and 1; [33]) was calculated to provide an estimate of the accuracy of rheotaxis. For a given orientation, high vector strength values indicate more accurate orientation towards that direction. In the WF condition, the latency for each tadpole to show stable rheotaxis, defined as the first of three consecutive 30 second sampling times in which the animal was oriented within +/-30° of 0°, was calculated. The 30° criterion for stable rheotaxis is the same metric used for quantifying stable rheotaxis in *X*. *laevis* tadpoles [13] and is within the range of angular deviations from 0° (10–45°) used to define rheotaxis in fishes [15, 18]. If a tadpole did not reach this criterion, then a latency value of 300 seconds was entered. The Kaplan-Meier estimator was used to calculate the probability at each 30 second time segment of a tadpole reaching a stable orientation, and a log-rank test was used to look for significant differences in latency between treated and untreated groups. This analysis is based on the assumption that once an individual has achieved a stable orientation according to the set criterion, that individual is considered to have maintained that stable orientation for the rest of the observation period, so that no individual can achieve a stable orientation more than once. Cox proportional hazards regression was used to test for the effects of treatment, flow speed and their interaction on latency.

Analyses of data from experiment 2 were conducted using GLM multivariate repeated measures ANOVA with treatment (2 levels, untreated and treated) and light location (2 levels, light upstream and light downstream) as between-subjects variables and flow (2 levels, NF and WF) as the repeated measure. XYZ coordinates are the multivariate dependent measures. Orientation headings were analyzed using circular statistics and the modified Rayleigh test.

## Results

### Experiment 1: Spatial positioning and orientation in the dark

Tadpoles responded to water flow by changing their XYZ position in the tank, and these responses differed in magnitude at different flow speeds. Results of multivariate analyses showed highly significant effects of flow [Pillai’s V = 0.579; F(3,255) = 116.9, P<0.0001] and of speed [Pillai’s V = 0.367; F(12,771) = 8.95, P<0.0001]. The interaction between flow and speed was also significant [Pillai’s V = 0.191; F(12,771) = 4.37, P<0.0001]. Univariate tests showed that flow affected positioning in all three dimensions, and that the interaction between flow and speed was driven by the X and Z dimensions. The main effect of treatment did not reach statistical significance (P = 0.164), suggesting that gentamicin did not disrupt swimming overall. There was a significant interaction between treatment and speed [Pillai’s V = 0.208; F(12,771) = 4.79, P< 0.0001], with univariate tests showing significant effects in all three dimensions. These results indicate that gentamicin affected spatial positioning in XYZ coordinates at some flow speeds.

**Fig 1:**
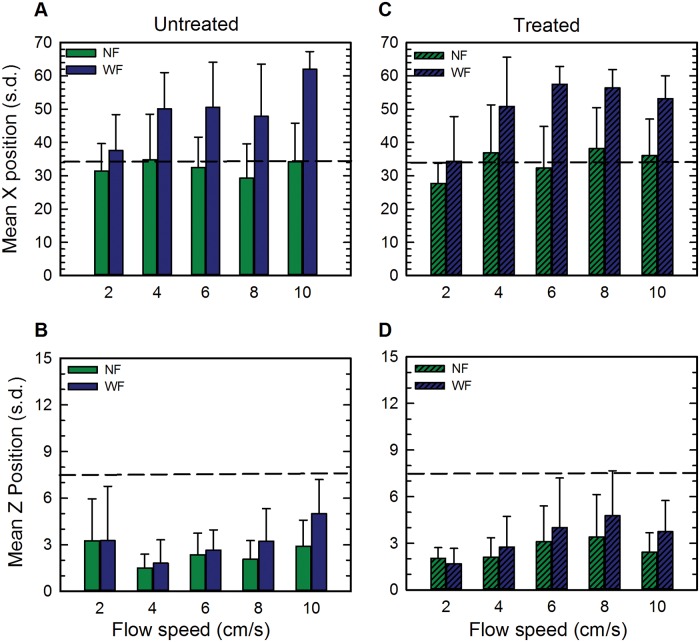
XZ spatial position at different flow speeds in Experiment 1 (tadpoles tested in the dark). (A) Untreated tadpoles, X position. (B) Untreated tadpoles, Z position. Solid green bars show position in NF (No Flow) and solid blue bars show position in WF (With Flow). (C) Treated tadpoles, X position. (D) Treated tadpoles, Z position. Hatched green bars show position in NF and hatched blue bars show position in WF. All positions are shown as mean +/- standard deviation. The horizontal dashed line on each plot shows the midpoint of the tank in that dimension. Both untreated and treated tadpoles move towards the back of the tank in the X dimension (A,C) and farther up in the water column in the Z dimension (B,D).

**Fig 2:**
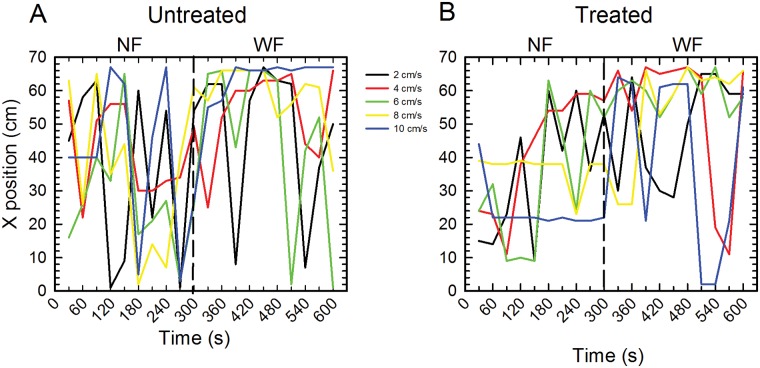
Changes in X position of individual tadpoles during NF and WF periods. (A) Untreated tadpoles. (B) Treated tadpoles. The NF period extends from 30 to 300 seconds and the WF period extends from 330 to 600 seconds. Flow is turned on at 300 seconds (vertical dashed line on each plot). Each colored line shows data from one tadpole at one flow speed. Tadpoles do not station hold in NF or in WF. Streamwise movements were reduced at a flow speed of 10 cm/s although crosswise movements still occurred. Tadpoles initiated movements within the first 30 second time interval after the flow is turned on, and change in movements were similar for untreated and treated tadpoles.

**Fig 3:**
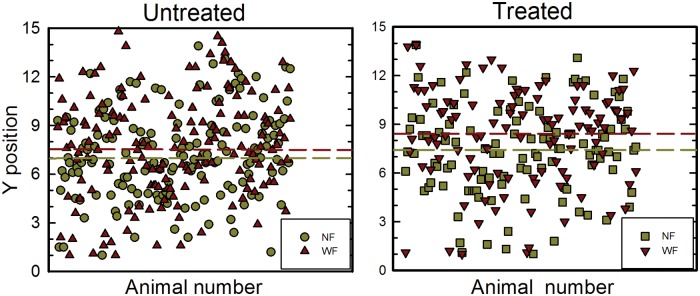
Y positioning of individual tadpoles during NF and WF periods. (A) Untreated tadpoles. Each data point shows the mean Y position of one tadpole over the NF period (dark yellow circles) and over the WF period (dark red triangles). Data are not separated by flow speed, because data points overlapped substantially. Over all tadpoles, the mean Y position in NF is 7.2 and the mean Y position in WF is 7.6, as shown by the color-coded dashed lines. (B) Treated tadpoles. Data in NF are shown as dark yellow squares and data in WF are shown as dark red inverted triangles. The mean Y position in NF is 7.5 and the mean Y position in WF is 8.3, as shown by the color-coded dashed lines. Tadpoles are more clustered towards the middle of the tank in NF than in WF, with treated tadpoles positioned farther towards one side in WF.

Positioning of untreated and treated tadpoles in XZ dimensions at all five flow speeds is shown in Fig 1. Individual variability in X positioning is displayed in Fig 2 and Y dimension data are shown in Fig 3. Untreated tadpoles (Fig 1A) in NF remained on average near the middle of the tank in the X dimension, but in WF, they swam downstream, towards the back of the tank. The mean magnitude of these downstream movements was 6.2 cm at 2 cm/s, 15.2 cm at 4 cm/s, 18.2 cm at 6 cm/s, 18.6 cm at 8 cm/s, and 27.8 cm at 10 cm/s. Downstream movements increased non-monotonically with flow speed, being least at 2 cm/s, similar in magnitude at speeds of 4, 6 and 8 cm/s, and greatest at 10 cm/s. Changes in X positioning of representative individual tadpoles at each flow speed at each of the 20 sampling times are displayed in Fig 2A. In NF, there was considerable variability in tadpole positioning, with most tadpoles swimming intermittently crosswise and streamwise with trajectories that varied between individuals. In WF, even though most tadpoles swam downstream, they did not station hold, but instead swam intermittently both streamwise and crosswise. Wall-following behaviors were not prominent in either flow condition.

In the Z dimension (Fig 1B), untreated tadpoles in NF were located near the bottom of the tank (Z positions of 1–3 cm). In response to flow speeds of 4cm/s and faster, tadpoles moved higher in the water column (mean magnitude of the response: 0.3 cm at 4 cm/s; 0.3 cm at 6 cm/s; 1.1 cm at 8 cm/s; 2.1 cm at 10 cm/s). At 2 cm/s, tadpoles remained on or near the bottom (mean change of -0.4 cm). Overall, changes in X and Z positioning in response to flow at 2 cm/s were smaller in magnitude and those at 10 cm/s were greater in magnitude than those at the other flow speeds (Tukey HSD, P<0.0001). These data suggest that tadpoles detected the flow and increased their responses, albeit non-monotonically, as flow speed increased.

Treated tadpoles (Fig 1C and 1D) showed similar positioning as untreated tadpoles. In the X dimension (Fig 1C), treated tadpoles swam downstream in WF, with the magnitudes of these positioning changes similar to those of untreated tadpoles at flow speeds of 2, 4, 6 and 8 cm/s (6.6 cm at 2 cm/s; 13.9 cm at 4 cm/s; 25.1 cm at 6 cm/s; 18.2 cm at 8 cm/s) but less extensive (17 cm) at 10 cm/s. In the Z dimension (Fig 1D), treated tadpoles in WF (at flow speeds of 4 cm/s and faster) tended to swim higher in the water column than they did in NF. The mean magnitudes of these changes are 0.6 cm at 4 cm/s; 0.9 cm at 6 cm/s; 1.3 cm at 8 cm/s; and 1.3 cm at 10 cm/s. Similar to untreated tadpoles, treated tadpoles did not station hold, but swam intermittently in the tank in both streamwise (Fig 2B) and crosswise dimensions.

Positioning in the Y dimension is displayed in Fig 3 as scatterplots; showing these data as mean Y position across animals obscures the skewed distribution of individual positions near one of the side walls. Untreated tadpoles in NF (Fig 3A) were located throughout the tank. In WF, these tadpoles tended to swim closer to one of the side walls. The mean magnitudes of changes in position were 0.4 cm at 2 cm/s; 0.7 cm at 4 cm/s; -0.2 cm at 6 cm/s; 1.3 cm at 8 cm/s; 0.2 cm at 10 cm/s). The overall pattern of Y positioning was similar in treated tadpoles (Fig 3B). The mean magnitudes of these movements varied somewhat with flow speed (0.9 cm at 2 cm/s; 1.9 cm at 4 cm/s; 0.08 cm at 6 cm/s; 0.4 cm at 8 cm/s; 1 cm at 10 cm/s), but were overall similar to those observed in untreated tadpoles.

**Fig 4:**
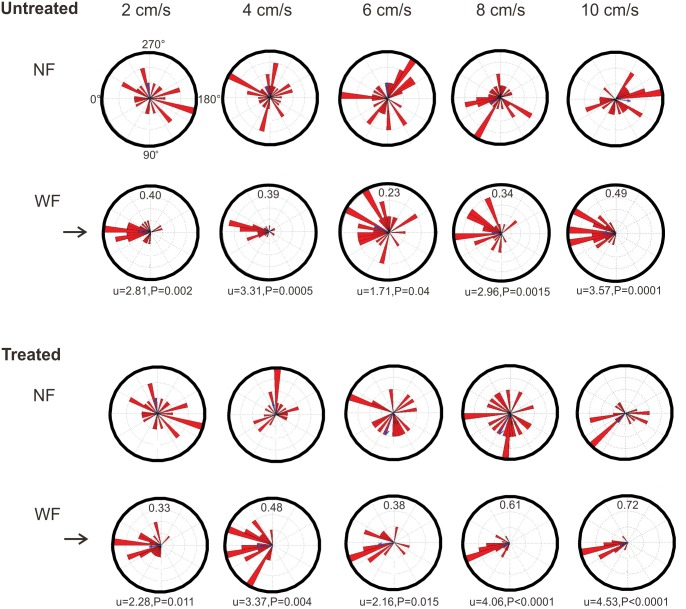
Orientation headings at different flow speeds in Experiment 1 (tadpoles tested in the dark). Circular plots (in degrees) showing orientation headings for untreated (A) and treated (B) tadpoles tested in NF (top row) and WF (bottom row) at different flow speeds (columns). The top NF plot (untreated, 2cm/s) shows the circular reference points (in degrees). The arrows on the left of the WF plots point to 0°, the crosswise midpoint of the source of the flow. An animal showing perfect positive rheotaxis would be oriented towards 0°. Red triangles (bin width of 5°) in each plot show the mean orientation, summed over ten time intervals (300 second total sampling time), of each individual tadpole. The length of the triangles indicates how many individual tadpoles exhibited that particular orientation. The numbers inside the circular plots in WF are the vector strengths of the orientation response. Results of the modified Rayleigh test (u, with corresponding P values) are shown below these plots. P values of 0.001 or below are statistically significant, according to our criterion. Statistical significance was not obtained in any NF condition.

Circular plots of orientation headings in both NF and WF periods and for untreated and treated tadpoles are shown in Fig 4. Untreated tadpoles (Fig 4A) were randomly oriented in NF, with a mean vector strength across “flow speeds” (within the 300 second NF period) of 0.06 (range 0.02 to 0.07). In WF, tadpoles tended to orient towards the source of the flow, but the value of the vector strength exceeded our criterion for statistical significance (P = 0.001) only at flow speeds of 4 cm/s and 10 cm/s. At 2 cm/s and 8 cm/s, vector strengths approached statistical significance (P = 0.002 and 0.0015), while at 6 cm/s, vector strength was smaller and not statistically significant (P = 0.04). The mean vector strength across all flow speeds was 0.37, indicating moderate accuracy of rheotaxis. At all flow speeds, orientation headings for individual tadpoles were dispersed, showing considerable inter-individual variability. Mean latency to achieve stable rheotaxis (orientation within 30° of 0° for three consecutive 30 second sampling times) ranged from 172 to 232 seconds at different flow speeds (Table 1). Even when achieving this criterion, only 16% of tadpoles remained oriented throughout the remainder of the WF period; most tadpoles changed their headings, as expected from their intermittent swimming.

**Table 1:** Stability of rheotaxis in Experiment 1 (tadpoles tested in the dark).

Group	Flow speed (cm/sec)	N	N (%) reaching criterion	N (%) not reaching criterion	Mean Latency* (s.e.)
**Untreated**	2	25	14 (56%)	11 (44%)	193.2 (22.1)
	4	36	22 (61%)	14 (39%)	172.5 (19.1)
	6	32	12 (38%)	20 (62%)	224.1 (19.5)
	8	38	14 (37%)	24 (63%)	222.6 (17.6)
	10	27	8 (30%)	19 (70%)	232.2 (21.3)
**Treated**	2	25	8 (32%)	17 (68%)	259.2 (14.3)
	4	24	10 (42%)	14 (57%)	218.8 (22.4)
	6	16	8 (50%)	8 (50%)	187.1 (29.8)
	8	25	12 (48%)	13 (52%)	216 (20.8)
	10	20	14 (70%)	6 (30%)	156.0 (26.5)

N = number of animals.

* A tadpole not meeting the criterion for stable rheotaxis was given a latency of 300 seconds, which was included in the calculations of latency.

s.e.: standard error of the mean

Treated tadpoles (Fig 4B), like untreated tadpoles, were randomly oriented in NF. In WF, treated tadpoles showed significant positive rheotaxis at fast flow speeds of 8 cm/s and 10 cm/s. Rheotaxis approached significance at slower flow speeds. Vector strength of the response ranged from 0.33 to 0.70, with a mean of 0.50, indicating moderate accuracy of orientation. Vector strengths were highest at the fastest flow speeds of 8 cm/s and 10 cm/s. Latencies for achieving stable rheotaxis varied from 156 to 260 seconds. At any given flow speed, orientation headings of individual tadpoles were variable, and across all speeds, 48% of treated tadpoles compared to 45% of untreated tadpoles met the criterion for stable rheotaxis. Mean latencies for meeting this criterion were 93 seconds for untreated tadpoles and 112 seconds for treated tadpoles, which are not statistically different (Kaplan-Meier estimator; this test excludes latency values for animals not reaching criterion). Once this criterion was reached, only 20% of treated tadpoles maintained their heading with 30° of the flow source. Results of the Cox proportional hazards regression showed a significant effect of treatment (hazard ratio = 0.38, P = 0.048). At a flow speed of 2 cm/s, treated tadpoles were significantly less likely to reach a stable orientation than untreated tadpoles; however, at a flow speed of 10 cm/s, treated tadpoles were more likely than untreated tadpoles to reach a stable orientation. These data suggest that the propensity for tadpoles to achieve stable rheotaxis is a function of both treatment and flow speed.

### Experiment 2: Spatial positioning and orientation in the presence of a light cue

**Fig 5:**
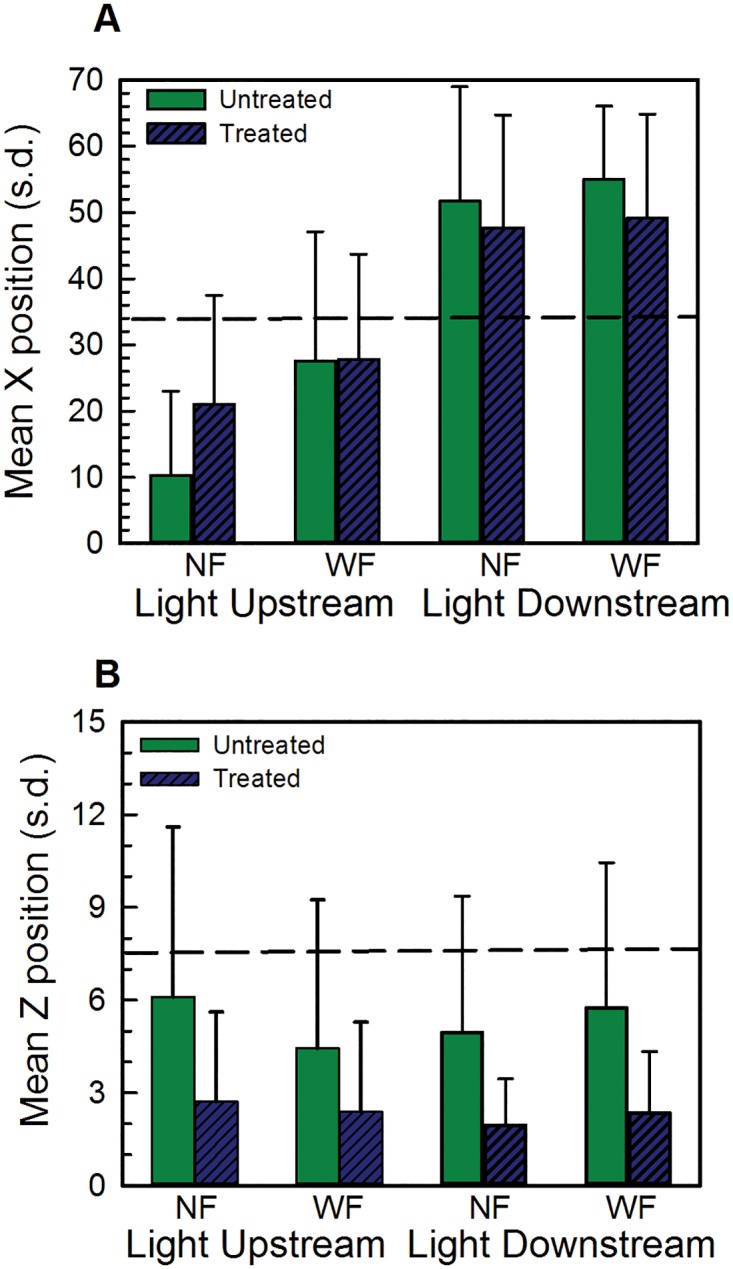
Spatial positioning in XZ dimensions in Experiment 2 (testing in the presence of a light cue). (A) Mean (+/- standard deviation) X positioning. (B) Mean (+/- standard deviation) Z positioning. Light location (upstream or downstream) and flow condition (NF, WF) are indicated on the x axis. In all plots, the dashed line shows the midpoint of the tank in that dimension. Untreated tadpoles are shown by the dark green bars and treated tadpoles are shown by the hatched dark blue bars. The presence and location of the light cue strongly affects positioning in the X dimension, while treatment affects positioning in the Z dimension.

The presence and location of a discrete light cue affected tadpoles’ positioning in the tank (Fig 5). Results of multivariate analyses showed significant effects of light position [Pillai’s V = 0.691; F(3,46) 34.34, P< 0.0001], treatment [Pillai’s V = 0.424; F(3,46) = 11.29, P< 0.0001], and flow [Pillai’s V = 0.379; F(3,46) = 9.34, P< 0.0001]. The interaction between flow and light location was also significant [Pillai’s V = 0.291; F(3,46) = 9.34, P = 0.001]. Results of univariate tests showed that light location affected X positioning [F(1,48) = 105, P<0.001] and that treatment affected Z positioning [F(1,48) = 33.87, P<0.001]. In NF, untreated tadpoles exhibited positive phototaxis. They were positioned in the X dimension close to the location of the light (Fig 5A), and were farther upstream when the light was located upstream and they were farther downstream when the light was located downstream. In WF, untreated tadpoles moved farther downstream, regardless of light location; however, tadpoles in the Light Upstream condition moved farther downstream (mean of 17.3 cm) than tadpoles in the Light Downstream condition (mean of 3.3 cm), or tadpoles tested in the dark (mean of 6.2 cm, Experiment 1). Behaviors of treated tadpoles varied with light location. In the Light Upstream condition, treated tadpoles in NF were located closer to the center of the tank (mean position 21 cm) than untreated tadpoles (mean position 10.2 cm; Fig 5A); in WF, X positioning of untreated and treated tadpoles did not differ (means of 27 and 28 cm), indicating that treated tadpoles did not move as far downstream. In the Light Downstream condition, treated tadpoles were located close to the position of untreated tadpoles in both NF and WF.

In the Z dimension (Fig 5B), untreated tadpoles in both the Light Upstream and the Light Downstream conditions in NF were located higher in the water column (means of 6.1 and 4.9 cm, respectively) than were untreated tadpoles tested in the dark (mean of 3.7 cm; Fig 1B). In WF, untreated tadpoles tested in the presence of light were positioned higher in the water column than untreated tadpoles tested in the dark (means of 4.4, 5.7 and 3.3 cm, respectively). In both light conditions, treated tadpoles were positioned lower in the water column than untreated tadpoles, both in NF and in WF.

Light location and gentamicin treatment did not appreciably affect position in the Y dimension. Both untreated and treated tadpoles were located throughout the tank in both NF and WF, and this positioning did not vary significantly depending on the location of the light cue.

**Fig 6:**
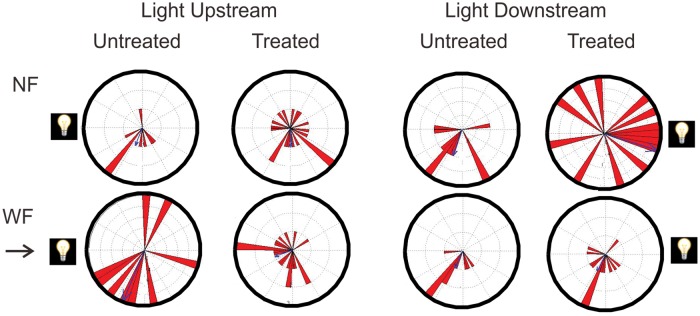
Orientation headings in Experiment 2 (testing in the presence of a light cue). Circular plots showing orientation headings of tadpoles tested in NF (top row) and WF (bottom row) in two light conditions (columns). Data are shown separately for untreated and treated animals. Tadpoles were not significantly oriented towards the location of the light cue (shown by the light symbol) or towards the source of the flow (in WF).

The presence of the light cue disrupted positive rheotaxis in both untreated and treated tadpoles (Fig 6), regardless of whether the light was located upstream or downstream. In NF, both untreated and treated tadpoles were randomly oriented, regardless of the location of the light cue. That is, tadpoles were not oriented towards the location of the light when no flow was present, and none of the vector strength values reached statistical significance. In WF, neither untreated nor treated tadpoles showed significant orientation either towards the source of the flow or towards the location of the light. In contrast, untreated tadpoles tested in the dark showed a strong trend towards positive rheotaxis (Fig 4A). These data suggest that the presence of a light cue disrupts rheotaxis.

### Visualization of neuromasts

**Fig 7:**
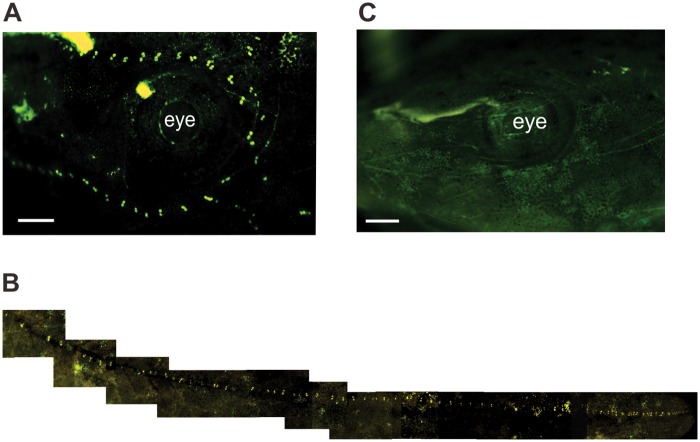
DASPEI-stained neuromasts in bullfrog tadpoles. (A) Supra- and infra-orbital lines in an untreated stage 26 tadpole. Rostral is to the right. Scale bar = 1mm. (B) Composite of images from the tail of an untreated stage 25 tadpole. The trunk is to the left and the tip of the tail is to the right. (C) Supra- and infraorbital lines in a gentamicin-treated stage 26 tadpole. In all images, DASPEI fluoresces yellow. Images have been adjusted for brightness, contrast and color balance.

DASPEI label of neuromasts was quantified in 7 untreated and 6 treated tadpoles. Images of stained neuromasts from untreated tadpoles are shown in Fig 7. Consistent with earlier descriptions [4], neuromasts were visible in oral, supraorbital, and infraorbital lines on the head, and dorsal, middle and ventral lines on the body. Oral neuromasts extended to the ventral surface of the body. Mean number of neuromasts in our sample was 175 (s.d.77), an average of 2.7 per mm body (head, trunk, and tail) length. This mean number is lower than that previously reported [4], but because that study did not report body length, it is not possible to compare neuromast density. Differences in counts may also reflect the transient nature of DASPEI staining—neuromasts could have lost their fluorescence over the time needed for visualization, photography, and quantification. Gentamicin treatment resulted in lack of DASPEI uptake. Mean number of stained neuromasts in treated tadpoles was 4 (s.d. 9).

## Discussion

Early larval bullfrog tadpoles show two different responses to current flow—they change their spatial position in XYZ coordinates and they change their orientation with respect to the flow source. These responses differ in strength and direction depending on the flow speed, and they are affected by the presence of a discrete light cue in an otherwise dark environment and by treatment with the ototoxin gentamicin. The pattern of behavioral changes with current flow, presence of light, and under conditions of gentamicin administration share both similarities and differences with flow-sensing behaviors of bullfrogs tested in a different flow field [14] and with those of larval *X*. *laevis* tested in an identical flow field [13]. Overall, the data show that rheotaxis is not a stereotyped behavior in larval anurans, but varies with environmental conditions and the structural integrity of the lateral line system.

### Spatial positioning in flow

In NF, untreated bullfrog tadpoles tested in the dark were as a group randomly positioned throughout the tank in the X (streamwise) dimension, distributed throughout the tank in the Y (crosswise) dimension, and located on or near the bottom of the tank (Z dimension). Tadpoles swam intermittently around the tank, with periods of movement punctuated by periods of position holding, but did not exhibit wall-following. In response to flow, tadpoles moved downstream, somewhat closer to a side wall, and higher in the water column. Changes in spatial position began during the first 30 seconds after flow onset, indicating rapid detection of flow. The magnitude of these changes varied with flow speed, suggesting that tadpoles could detect differences in flow and that flow effected changes in behavior. Tadpoles did not station hold in flow; instead, they swam intermittently both streamwise and crosswise, even while tending to remain within the downstream half of the tank. At the fastest flow speed of 10 cm/s, tadpoles tended to remain close to the downstream wall, an area of reduced flow due to boundary effects. Locations near side walls are also areas of reduced flow. These data suggest that tadpoles prefer locations where flow is minimized, particularly when challenged with fast flow speeds.

Downstream positioning after the onset of flow might suggest that tadpoles did not actively swim downstream or searching out areas of reduced flow, but rather were passively displaced by the current. Although such an effect cannot be completely ruled out, especially at fast flow speeds, changes in the magnitude of downstream movements with increases in flow speed were not monotonic, indicating that these movements were not simply the result of passive displacement. Moreover, the absence of station holding and the presence of counterflow swimming at all flow speeds indicate that tadpoles were actively moving around the tank.

Changes in spatial positioning seen here are consistent with those reported previously [14] in a study of bullfrog tadpoles in the same larval stages tested in a less homogenous flow field and under red rather than infrared light. In that study, tadpoles also swam downstream in response to current flow (speeds of 6, 8, and 10 cm/s), and preferred positions near the side walls and on or near the tank bottom. Tadpoles did not station hold; instead, even at the fastest flow speeds, they tended to swim crosswise or partially upstream while remaining in the downstream half of the tank. Comparisons between the two studies suggest that the specific dynamics of the flow field and the source of illumination did not appreciably affect XY positioning in flow. Differences were present in the Z dimension; in the current study, tadpoles tended to swim higher in the water column in WF, while still remaining in the lower third of the tank’s depth; in the previous study, tadpoles tended to remain on the bottom of the tank, on average about 1 cm lower. These differences may reflect variability between animals or the presence of a larger boundary zone in the earlier study.

In spite of these general trends, analyses of individual movement trajectories in both studies show considerable inter-individual variability in spatial positioning. There is no evidence that there was a unique location in the tank preferred by all tadpoles, either in NF or WF, that could have been produced by some extraneous cue. Schmidt et al. [14] suggested that the presence of individual variability indicates that tadpoles behave consistent with a model based on a directed random walk. In such a model, spatial positioning is selected randomly from a probability distribution, with the walker selecting a heading at random and then following that heading for some interval, before changing again at random. The onset of flow might shift the starting position downstream, through some combination of passive displacement and an active behavioral response. The walker would then select a new position (upstream or downstream) from a probability distribution, with changes in positioning operating as before.

Spatial positioning in flow by bullfrog tadpoles show both similarities and differences with that observed in larval *X*. *laevis* [13] tested at equivalent early larval stages, in the same flow field, and at the same flow speeds (2 and 4 cm/s). *X*. *laevis* tadpoles also swam downstream in response to flow, with a mean change in position of 12.8 cm, not statistically different from the 11.6 cm change observed in the present study. At the new X position, these tadpoles, unlike bullfrogs, exhibited stable station holding, with few additional streamwise or crosswise movements. Movements in WF in the Y dimension differ somewhat but not significantly (*X*. *laevis*: -0.05 cm, indicating small movements closer to the crosswise midpoint; bullfrogs: 0.58cm, small movements towards a side wall). Changes in Z positioning vary significantly [t(113) = -3.09, P = 0.003]. *X*. *laevis* tadpoles hung suspended in the water column (hydrostatic balancing mechanism; [34]) in NF, and moved towards the bottom on the tank in WF (mean change of -1.6 cm), while bullfrog tadpoles remained on or near the bottom of the tank in NF, and swam slightly upwards (mean change of 0.02 cm) in WF.

### Orientation and rheotaxis

A major result of this study is that bullfrog tadpoles can exhibit rheotaxis under some testing conditions; however, the propensity to show rheotaxis varied considerably across individuals, the accuracy of rheotaxis was moderate, and latency to achieve stable rheotaxis was long. Untreated tadpoles tested in the dark were randomly oriented in NF, as expected, but exhibited positive rheotaxis (near or exceeding statistical significance) at four of the five flow speeds. Across all five flow speeds, between 30–61% of tadpoles met the criterion for stable rheotaxis, and then only after a mean latency (calculated using data only from animals that exhibited rheotaxis) of 93 seconds. Accuracy of rheotaxis, estimated by the vector strength of the response, was greatest at 10cm/s, but differences between those at 2, 4, and 8 cm/s were not large, and accuracy did not change monotonically with flow speed. Schmidt et al. [14], studying the same species at the same larval stages, did not find significant positive rheotaxis at flow speeds of 6, 8 and 10 cm/s (slower flow speeds were not tested in that experiment). The major difference between these two studies is in the characteristics of the flow field, which was more homogeneous in the present work. These data show that bullfrog tadpoles are sensitive to flow dynamics, but exhibit rheotaxis with considerable variability.

**Fig 8:**
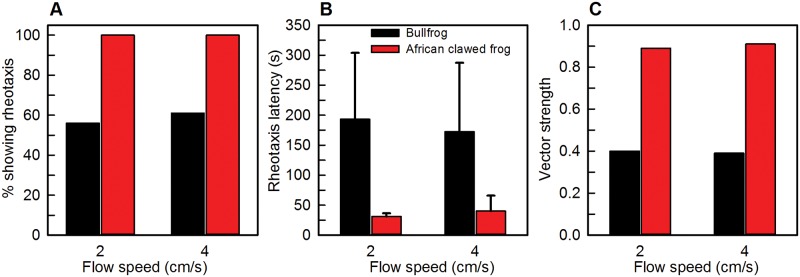
Comparison of rheotaxis behaviors in bullfrogs (*Rana catesbeiana*) and African clawed frogs (*Xenopus laevis*). Tadpoles at comparable developmental stages were tested in the same flow tank at flow speeds of 2 and 4 cm/s. Black bars show data from bullfrogs and red bars show data from African clawed frogs. (A) Percent of animals showing significant positive rheotaxis. At both flow speeds, all African clawed frog tadpoles show rheotaxis, compared to a mean of 58% of bullfrog tadpoles. (B) Latency to achieve rheotaxis is longer in bullfrog tadpoles (mean of 183 seconds; including responses of animals that did not reach the criterion for rheotaxis) than in African clawed frog tadpoles (mean of 37 seconds). (C) Vector strength of the orientation response is higher in African clawed frog tadpoles (mean of 0.90) compared to bullfrog tadpoles (mean of 0.40).

In contrast, *X*. *laevis* tadpoles, at developmental stages from early larval to metamorphic climax, tested in the same flow field showed robust, accurate positive rheotaxis at all flow speeds tested [13]. Comparative data from untreated bullfrogs and *X*. *laevis* tested at comparable early larval stages, at the same flow speeds of 2 and 4cm/s, and in the dark are shown in Fig 8. These comparisons show that rheotaxis is a more stereotyped and prominent behavior in larval *X*. *laevis* than in larval bullfrogs.

In fishes with a functioning lateral line system, accuracy of rheotaxis tends to be greater at faster compared to slower flow speeds [15, 17, 18, 26, 35]. Such a trend is not seen in larval anurans. In both bullfrogs (this study) and *X*. *laevis* [13], rheotaxis shows similar accuracy and variability at slow and fast flow speeds. Comparisons of data from tadpoles and fishes are difficult due to differences in experimental variables such as the nature of the flow source (radial or laminar flow), different criteria for rheotaxis, and whether orientation is based on headings of individual animals or calculated as a proportion from group data. In addition, many studies do not present a visual display of the flow field, so it is not possible to determine how any differences in flow dynamics between studies may have affected the fishes’ behavioral responses.

Bak-Coleman and Coombs [27] examined XY spatial positioning and rheotaxis in two fish species, one classified as sedentary and benthic (three-lined corydoras, *Corydoras trilineatus*) and another classified as mobile (Mexican blind cave fish, *Astyanax mexicanus*). Fishes were exposed to flow in the dark in a tank of similar design to that used in this study. Both species moved upstream in response to fast flow speeds. At low flow speeds, the sedentary species preferred the downstream portion of the tank. The mobile species exhibited wall-following behaviors at all except the fastest flow speeds while the sedentary species did not exhibit wall-following behaviors. These two species also differed in rheotaxis. Sedentary *C*. *trilineatus* exhibited rheotaxis at fast flow speeds but not at slow flow speeds, and this behavior was disrupted by treatment with an ototoxin. In contrast, mobile *A*. *mexicanus* showed similar accuracy of rheotaxis before and after ototoxin treatment. The authors suggested that these behavioral differences and the importance of lateral line compared to other cues in orientation reflect the degree to which the fish were “coupled” to the substrate [27]. Bullfrog and *X*. *laevis* tadpoles are more sedentary than they are mobile. Instead of swimming continuously, both species move in short bouts punctuated by sedentary behaviors [36]. Neither displays wall-following behaviors and neither moves far upstream in response to fast current flow. Yet, they differ in the strength and accuracy of rheotaxis. Distinctions based on movement patterns do not appear to be useful for explaining variability of positioning and orientation behaviors in larval anurans.

### Effects of light on positioning and rheotaxis

The presence of a discrete light cue in an otherwise dark testing environment altered bullfrog tadpoles’ spatial positioning and orientation. Untreated tadpoles in NF exhibited positive phototaxis—they swam farther upstream in the Light Upstream condition and farther downstream in the Light Downstream condition, and they were positioned higher in the water column. In response to flow, they swam downstream away from the light and closer to the bottom of the tank. That is, positive phototaxis was reversed by current flow. The presence of the light also disrupted rheotaxis. Tadpoles did not orient towards the source of the flow nor towards the location of the light in either Light Upstream or Light Downstream conditions, even though they were faced with the same flow speed that produced rheotaxis in tadpoles tested in the dark. These data are consistent with results in *X*. *laevis* tadpoles [13], whose spatial positioning and rheotaxis are also altered by the presence of a discrete light cue. Even though the light cues are visible to the tadpoles, as shown by positive phototaxis in the absence of flow, and so could provide a fixed external reference frame for orientation, they do not guide positive rheotaxis. This suggests that lateral line cues are predominant over visual cues.

In fishes, the influence of the visual environment on rheotaxis has been examined by comparing orientation under diffuse visible light and in a dark (red or infrared) environment. Rheotaxis by the diurnal species the giant danio (*Devario aequipinnatus*) is more precise under visible than under infrared light [18], suggesting that vision can guide orientation and may be more salient than lateral line cues. Results from larval zebrafish are variable, with one study showing similar accuracy of rheotaxis in visible and dark conditions [16] and another study showing less accurate rheotaxis under diffuse illumination [17]. In *X*. *laevis* tadpoles, rheotaxis is similar under diffuse and dark (red) illumination [12], suggesting that visual cues are not primary for guiding orientation in this species. The presence of discrete light cues disrupts rheotaxis in both *X*. *laevis* [13] and in bullfrogs (this study). These data suggest that tadpoles privilege lateral line over visual cues for orientation in the presence of current flow. It is not known how a discrete light source, as used in this study, would affect rheotaxis in fishes.

### Effects of gentamicin on positioning and rheotaxis

Gentamicin treatment affected both spatial positioning and rheotaxis of bullfrog tadpoles, but did not completely disrupt these behaviors. For tadpoles tested in the dark, there was no evidence that gentamicin disrupted motor function; intermittent swimming still occurred, wall-following and station holding did not emerge, and spatial positioning in NF was similar to those of untreated tadpoles. In WF, treated tadpoles tended to swim higher in the water column than untreated tadpoles and showed less extensive downstream movements at the fastest flow speed. These data suggest that gentamicin affected the tadpole’s ability to sense areas in the tank where flow was minimized. Gentamicin did not disrupt rheotaxis. At the slowest flow speed tested, rheotaxis was less accurate in treated than in untreated tadpoles, but at the fastest flow speed, rheotaxis was more accurate in treated tadpoles. Accurate rheotaxis at fast flow speeds has also been observed in gentamicin-treated *A*. *mexicanus* [26]. Treated tadpoles showed considerable individual variability in orientation headings and achieved stable rheotaxis only after long latencies. Even at a flow speed of 10 cm/s, 30% of treated tadpoles did not meet the criterion for stable rheotaxis, and only 20% of tadpoles that met the criterion maintained their orientation towards the flow throughout the WF period. Thus, although rheotaxis was statistically more robust in treated tadpoles, especially at fast flow speeds, the behavior did not become more stereotyped.

There are two possible explanations for this pattern of results. One is that gentamicin may not have been effective in disrupting neuromast function in tadpoles. In fishes, rheotaxis is controlled by superficial neuromasts, and there are conflicting data on whether gentamicin treatment damages these neuromasts [15, 20, 21, 35, 37,38]. The same dosage of gentamicin used in this study produced neuromast damage in larval zebrafish [21] and disrupted but did not eliminate rheotaxis in larval *X*. *laevis* [13]. In both bullfrog and *X*. *laevis* tadpoles, we observed less DASPEI label in treated compared to untreated tadpoles. These data suggest that the gentamicin treatment did damage neuromasts; however, because we did not visualize DASPEI label in all treated tadpoles, we cannot rule out the possibility that gentamicin’s actions varied between individual animals, and that some animals had enough residual neuromasts to mediate rheotaxis.

Another possible explanation for the behavioral data is that tadpoles may have relied on proprioceptive and/or vestibular cues to mediate orientation under conditions of lateral line damage, particularly when faced with fast current flows. Bullfrog tadpoles are benthic and could derive proprioceptive cues from the substrate to sense their movement relative to water flow. In addition, even in early larval stages, vestibular organs in the inner ear contain hair cells, are innervated by nerve fibers, and contribute to central processing of particle motion cues [24]. The behavioral salience of proprioceptive or vestibular cues for controlling flow sensing has not been explicitly examined.

In bullfrog tadpoles, gentamicin did not reverse or reduce the disruption of rheotaxis produced by the presence of a discrete light cue. Similar results were found for larval *X*. *laevis* [13]. Thus, there is no evidence in these animals that visual cues can compensate or substitute for a damaged lateral line, as has been proposed in fishes [1, 15, 18, 19, 26]. It is not known if diffuse visible light would facilitate rheotaxis in treated tadpoles, however.

### Variability of lateral line function in tadpoles

On the basis of internal and external morphology and mode of feeding, tadpoles have been classified into four basic ecomorphological types [23]. *X*. *laevis*, a midwater suspension feeder that does not become benthic until metamorphic climax, is Type 1 and *R*. *catesbeiana*, a benthic and lentic “generalized pond form,” [4] is Type 4. Results of behavioral experiments (this study and [13]), show that these two species, tested in the same flow field under identical speed and lighting conditions, do not exhibit comparable rheotaxis. This is in spite of the biological importance for detection of changes in background current flow, for detecting the approach of a swimming predator or the presence of a prey item (floating plant matter or detritus), for example. It is important to consider aspects of the biology of these two species that could contribute to these behavioral differences.

A primary difference is in the morphology of the lateral line neuromasts [4, 5]. Lannoo [4] described neuromasts of bullfrog tadpoles as small (containing less than 15 hair cells), and arranged in linear stitches with fewer than five neuromasts. In *X*. *laevis*, neuromasts were described as large (containing more than 15 hair cells) and arranged in loosely clumped stitches with more than five neuromasts. These data were based on only two tadpoles from each species, however, and then at only two larval stages, and so need to be confirmed with a larger sample. Our DASPEI data, also based on a limited sample, show that at comparable developmental stages, *X*. *laevis* tadpoles have more neuromasts (22.8 per mm of head/trunk/tail length) than do bullfrog tadpoles (2.7 per mm body/tail length). *X*. *laevis* tadpoles may thus exhibit more stereotyped rheotaxis because they have more sensors.

These two species differ in other behaviors that may be mediated at least partially by the lateral line system. One difference is in aggregation behaviors. *X*. *laevis* exhibits schooling behaviors that are similar to those of fishes [7, 36] and that are disrupted by aminoglycoside treatment [8]. Bullfrog tadpoles, on the other hand, do not school and are only loosely clumped, without precise alignment (personal observations and [36]). It is not known if lateral line cues play a role in this clumping arrangement. Another difference is in postures. Bullfrog tadpoles are benthic throughout development, while *X*. *laevis* up until metamorphic climax stages hang head downward in the water column. The relative degree to which these different postures depend on lateral line, proprioceptive, and vestibular input is not known. Because of similar trajectories in the development of the saccule and the semicircular canals in these two species [24], it is unlikely that the use of vestibular cues for sensing current or self-movement differs, but this has not been verified experimentally.

A final difference between these two species is in life history and the extent of morphological changes during metamorphosis. *X*. *laevis* remains aquatic after metamorphosis. Numbers of neuromasts increase throughout tadpole stages [13], and are maintained in juvenile and adult forms [10]. The lateral line may thus remain relevant for mediating orientation and other behaviors throughout the life span. In bullfrog tadpoles, on the other hand, neuromasts begin to degenerate at the beginning of metamorphic climax, coincident with the emergence of forelimbs, and are completely absent by the end of climax. During this crucial developmental stage, the animal must begin to rely on other sensory cues for orientation, navigation, and predator avoidance. Bullfrog tadpoles may have begun to integrate or to privilege input from other sensory systems even during earlier stages of development to prepare for the eventual loss of their lateral line system.
